# Duckweed’s Effects on Rice Yield and Quality Varied with Fertilizer Applications

**DOI:** 10.3390/plants14182850

**Published:** 2025-09-12

**Authors:** Yipeng Zhao, Guizhi Shi, Jingsheng Luo, Xinyong Zhao, Shaowu Hu, Tingting Hu, Lianxin Yang, Yunxia Wang

**Affiliations:** 1 College of Environmental Science and Engineering, Yangzhou University, Yangzhou 225009, China; zhaoyipeng@yeah.net (Y.Z.); mx120230728@stu.yzu.edu.cn (G.S.);; 2 Xuzhou Institute of Agricultural Science, Xuzhou 221131, China; zhaoxinyong2020@163.com (X.Z.); htt713@163.com (T.H.); 3 Key Laboratory of Crop Genetics and Physiology of Jiangsu Province, Co-Innovation Center for Modern Production Technology of Grain Crops of Jiangsu Province, Yangzhou University, Yangzhou 225009, Chinalxyang@yzu.edu.cn (L.Y.)

**Keywords:** *Oryza sativa* L., fertilizer management, *Lemna minor* L., grain quality

## Abstract

The incidence of duckweed (*Lemna minor* L.) outbreaks in paddy fields has increased in recent years, but how it impacts rice production is still under debate. This study assessed duckweed’s effects on rice yield and quality under different fertilizer regimes: organic fertilizer (OF), chemical fertilizer (CF), a mix (one-third OF and two-thirds CF based on nitrogen content, COF), and no fertilizer (NF) as a control. For each fertilizer regime, two duckweed treatments were applied: duckweed coverage (Duckweed) and no duckweed coverage (Control). A light wet–dry alternate irrigation method was used in the experimental field. Averaged across all fertilizer treatments, duckweed coverage in paddy fields increased grain yield by 8.3%, mainly due to increased panicle density. Duckweed coverage increased chalky grain percentage by 17.0% under NF, but decreased it by 33.7% under CF, with nonsignificant changes under COF and OF conditions. Similar fertilizer-by-duckweed interactions were also found for chalkiness degree, white degree, breakdown and setback values of the starch rapid visco analyzer (RVA) profile, palatability index, and protein and amino acid concentrations. Duckweed coverage decreased protein and amino acid concentrations but improved the taste of cooked rice under NF, while the opposite trend was observed under CF. Duckweed coverage significantly decreased copper and zinc concentrations in milled rice, which may aggravate the “hidden hunger” risk for rice consumers.

## 1. Introduction

As a small, free-floating aquatic plant, duckweed moves easily by water flow or wind and reproduces rapidly in favorable environments with proper temperature, abundant light, and sufficient nutrients [1]. Due to over-application of fertilizers and eutrophication of water bodies in areas experiencing rapid economic growth but less strict environmental regulation, duckweed outbreaks are common in irrigation ditches and paddy fields [2,3,4]. To date, the most studied aspect of duckweed–rice association is how duckweed affects nitrogen fertilizer efficiency in paddy fields [5,6,7]. Duckweed covers water surface, forms a physical barrier that blocks ammonia volatilization directly, or indirectly inhibits ammonia volatilization by lowering the water temperature and pH [8,9,10]. In terms of rice production, a few studies have monitored the effect of duckweed on rice yield, and only one preliminary study has examined its impact on rice quality [11].

Previous studies have found that duckweed mulching in paddy fields can increase [5,8,9], decrease [12], or have no change [6,13] on rice yield. A recent study suggested that different fertilizer applications could affect duckweed’s effects on rice yield: duckweed mulching significantly increased the rice yield with the application of urea or biogas slurry, but the yield increase was nonsignificant with the application of slow-release fertilizer or no fertilizer [10]. This may suggest that under conditions of nutrient sufficiency, duckweed occurrence in paddy fields is beneficial for rice production, while under conditions of nutrient deficiency, the beneficial effect is weakened by duckweed competing with rice for nutrients.

However, in our previous study trying to address the above hypothesis, where fertile lotus-pond bottom soil was used as a growth medium to secure sufficient nutrients for both duckweed and rice, no significant change in rice yield by duckweed was found for three rice cultivars [11]. Further analysis indicated trade-offs among yield components in response to duckweed coverage, namely, a decrease in the panicle density offsets the increase in the spikelet density and/or grain weight [11]. This study suggested that even if the macronutrient supply was secured, there was no guarantee of yield increases. Continued flooding of fields also caused reduced soil conditions. Further declines in soil redox potential due to duckweed decomposition induced micronutrient deficiency, especially of zinc and copper during the seedling stage, and severe micronutrient deficiency substantially inhibited rice tiller formation [11]. Therefore, different soil conditions imposed by various fertilizer strategies may be the cause of the complex rice-by-duckweed interactions observed previously [10]. The most recent report by Liu et al. [7] suggested that duckweed’s impact on rice growth and yield was also affected by irrigation methods: under alternate wet and dry irrigation, duckweed in paddy fields increased rice yield by increasing tillers, while under continuous flooding, duckweed caused a trend of decrease in tillers and yield.

The complex interactions between duckweed and rice during the rice growth season affect plant physiological and biochemical processes in filling grains, which will ultimately be reflected in changes to not only rice yield but also rice quality. In our preliminary study, some of the rice quality traits were measured, and the most striking change induced by the duckweed was the decreased micronutrient concentrations of copper and zinc in rice grains, despite the increased protein concentration [11]. This preliminary study suggested that duckweed coverage during rice growth changed rice nutrient uptake and grain nutritional quality under these circumstances. Whether similar impacts occur in paddy fields remains unclear; furthermore, is it possible to eliminate this negative effect of duckweed on grain nutrient quality through field management? The present study addresses these questions through the following approaches: 1, chemical and organic fertilizers with different dosage combinations were applied to the paddy field; 2, dry–wet irrigation cycles were employed throughout the entire rice season to induce frequent duckweed growth–decomposition cycles. The hypothesis is that duckweed’s effects on rice yield and quality will vary with fertilizer application regimes.

## 2. Results

### 2.1. Effect of Duckweed Coverage on Grain Yield Under Different Fertilizer Applications

Fertilizer application significantly increased rice yield (Table 1). Compared to only 5448.6 kg ha^−1^ under no fertilizer (NF) control, the grain yield of rice supplied with chemical fertilizer (CF), the mix of two-thirds of CF and one-third of organic fertilizer (COF), and organic fertilizer (OF) was 11,969.2 kg ha^−1^, 11,524.4 kg ha^−1^, and 9743.5 kg ha^−1^, respectively. Averaged across fertilizer treatments, duckweed coverage increased grain yield by 8.3%, but for each fertilizer treatment, the duckweed’s effect was statistically nonsignificant except for the NF treatment. No significant fertilizer-by-duckweed interaction was detected on rice yield (Table 1).

Both fertilizer application and duckweed coverage significantly increased panicle density (panicle number per m^2^). Duckweed coverage increased panicle density by 21.4%, 26.2%, and 33.3% under NF, COF, and OF conditions, respectively, but had no effect under CF, which led to significant fertilizer-by-duckweed interactions (Table 1). Neither fertilizer nor duckweed treatment changed the spikelet number per panicle. Filled-grain percentage and individual grain weight were affected by fertilizer but not by duckweed treatment. Compared with CF and COF, NF and OF had higher filled-grain percentages and individual grain weights (Table 1).

### 2.2. Effect of Duckweed Coverage on Grain Quality Under Different Fertilizer Applications

#### 2.2.1. Processing Quality

Neither fertilizer nor duckweed treatment had significant effects on brown rice percentage (Table 2). Milled rice percentage varied with fertilizer treatments: OF showed the highest milled rice percentage, followed by NF, CF, and COF. Duckweed coverage during rice growth reduced the milled rice percentage of the OF treatment, but its effect on the milled rice percentage of the other fertilizer treatments was nonsignificant (Table 2). On average, neither fertilizer nor duckweed treatment had a significant effect on head rice percentage, but a significant fertilizer-by-duckweed interaction indicated that the duckweed effect differed between fertilizer treatments (Table 2).

#### 2.2.2. Appearance Quality

The appearance of both brown rice (Table 3) and head rice (Table 4) was assessed. Different fertilizer applications affected grain maturity, with the weight ratio of immature grains being lower under NF and OF conditions than under CF and COF conditions (Table 3). Averaged across all fertilizer treatments, duckweed coverage increased the weight ratio of immature grains by 17.2%, and the significant increase was found for the plants supplied with OF. Compared with NF, fertilizer applications generally increased grain length and grain width but decreased the grain length–width ratio. Duckweed coverage increased grain length but did not change grain width, resulting in a higher grain length–width ratio (Table 3). Different fertilizer applications affected grain color, with the white degree being higher for NF and OF than CF and COF. An opposite trend was observed for Ym among different fertilizer treatments. Duckweed coverage had no significant effect on grain color (Table 3).

The grain shape of head rice in response to fertilizer treatments was similar to that of brown rice (Table 4). Compared with NF, fertilizer application generally increased the grain length and grain width of head rice. Except under NF conditions, duckweed coverage increased grain length and grain width, especially under CF conditions. Neither fertilizer nor duckweed treatment changed the grain length–width ratio. Compared to NF and OF, the head rice of CF and COF had much less grain chalkiness, as shown by the significant lower chalky grain percentage and chalkiness degree (Table 4). Significant fertilizer-by-duckweed interactions on chalky grain percentage indicated that duckweed effects varied with fertilizer treatments. Duckweed coverage increased chalky grain percentage by 17.0% under NF but decreased it by 33.7% under CF, while the decreases under COF and OF were nonsignificant. A similar trend of fertilizer-by-duckweed interaction was observed for chalkiness degree. The white degree of head rice under NF and OF conditions was higher than that under CF and COF conditions. On the contrary, Ym of head rice under NF and OF conditions was lower than that under CF and COF conditions. Significant fertilizer-by-duckweed interactions were found for both Ym and white degree of head rice (Table 4).

### 2.3. Cooking and Eating Quality

Fertilizer treatment significantly affected the amylose content of head rice (Table 5). The grains obtained from plants grown under the CF condition had the highest amylose content of 13.3%, while those under the NF condition had the lowest value of 11.1%, averaged across duckweed treatments. Averaged across all fertilizer treatments, duckweed had no significant effect on amylose content (Table 5).

The rapid visco analyzer (RVA) profile of milled rice flours was significantly affected by fertilizer treatments and fertilizer-by-duckweed interactions (Table 5, Figure S1). Averaged across duckweed treatments, the values of viscosities in the RVA profile were lower under CF and COF conditions than under NF and OF conditions. Duckweed’s effects on the RVA profile of milled rice varied with fertilizer applications, as shown by the different shifts in RVA profile among fertilizer treatments (Figure S1) and the interactions between duckweed and fertilizer treatments on the parameters of RVA (Table 5). Duckweed coverage decreased the breakdown value by 12.0% for the rice grown under the CF, but the changes in breakdown values for other fertilizer treatments were not significant. Duckweed coverage decreased the setback value by 29.7% for the rice grown under the NF but increased it by 19.6% under the CF, with no significant changes observed under other fertilizer treatments (Table 5). Duckweed coverage shortened peak time under NF but prolonged peak time under CF and had no significant effect on peak time under the other two fertilizer treatments. Duckweed coverage increased the pasting temperature for rice grown under OF, but the effect was nonsignificant under other fertilizer treatments (Table 5).

The overall palatability of cooked rice was significantly affected by the treatment of fertilizer, duckweed, and their interactions (Table 6). Averaged across two duckweed treatments, the overall palatability index was 75.7 and 75.2 under NF and OF, respectively, being higher than that under CF (67.0) and COF (65.8). Averaged across all fertilizer treatments, duckweed coverage increased the overall palatability index by 4.6%. Duckweed coverage increased the overall palatability index by 18.3% under NF but decreased it by 11.3% under CF and had no significant effect on overall palatability under COF and OF conditions.

The responses of luster, stickiness, and balance degree of cooked rice to fertilizer, duckweed, and their interaction were similar to those of the overall palatability index (Table 6). Duckweed coverage increased luster, stickiness, and balance degree under NF but decreased each of these parameters under CF and had no significant effect under COF and OF conditions. On the contrary, duckweed coverage decreased the hardness of cooked rice by 15.1% under NF but increased it by 11.8% under CF. In summary, chemical fertilizer application decreased the eating quality of cooked rice compared with no fertilizer or organic fertilizer application. Duckweed coverage improved eating quality under NF, decreased it under CF, and had no effect on it under OF (Table 6).

### 2.4. Nutritional Quality

**Figure 1:**
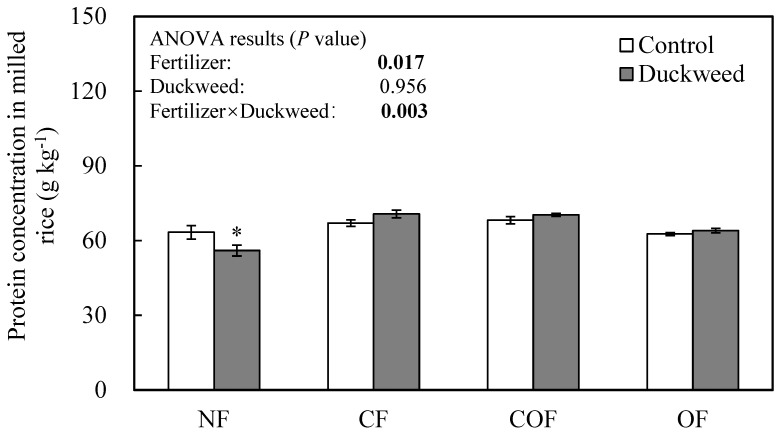
Effects of duckweed coverage on protein concentration of milled rice under different fertilization conditions. NF, no fertilizer application; CF, chemical fertilizer; COF, two-thirds of CF and one-third of organic fertilizer; OF, organic fertilizer. Control, no duckweed coverage; Duckweed, duckweed coverage. Each bar in the figure represents the mean value ± standard error. * indicates significant duckweed effect at *p* < 0.05. ANOVA values in bold indicate significance at *p* < 0.05.

Fertilizer treatment had a significant effect on the protein concentration in milled rice, with the lowest value of 59.7 g kg^−1^ in NF and the highest value of 63.3 g kg^−1^ in CF (Figure 1). Duckweed coverage significantly decreased protein concentration by 11.6% under NF conditions but tended to increase protein concentrations under CF, COF, and OF conditions (Figure 1).

Similar trends of fertilizer, duckweed, and their interactions were found for the concentration of essential amino acids, non-essential amino acids, and total amino acids (Table 7). The concentrations of these three groups of amino acids in rice supplied with CF and COF were higher than those with OF and NF. Duckweed coverage significantly decreased the concentration of essential amino acids, non-essential amino acids, and total amino acids by 11.3%, 11.9%, and 11.7%, respectively, under NF conditions, but increasing trends were observed under CF and OF conditions (Table 7).

In general, the concentrations of individual amino acids of milled rice in response to fertilizer, duckweed, and their interaction were similar to those of amino acid groups or protein (Table 8). The concentrations of individual amino acids were decreased by duckweed coverage under NF but increased under CF or OF. Significant fertilizer-by-duckweed interactions were found for the concentration of leucine, lysine, glutamate, glycine, alanine, and histidine (Table 8).

Duckweed coverage had no effect on the concentration of calcium, potassium, magnesium, phosphorus, iron, or manganese in milled rice but significantly decreased the copper concentration by 30.1% and zinc concentration by 16.2% when averaged across the four fertilizer treatments (Table 9). Zinc concentration reductions by duckweed varied with different fertilizer applications, with decreases of 21.0%, 14.0%, 14.9%, and 13.2% under NF, CF, COF, and OF conditions, respectively. Compared with NF, zinc concentrations in milled rice were lower under fertilizer applications (Table 9).

## 3. Discussion

### 3.1. Fertilizer Application on Rice Yield: Organic Fertilizer vs. Chemical Fertilizer

In order to secure high crop yield, the past several decades have seen increasing chemical fertilizer input to farmland, which has caused several problems: soil structure damage, soil nutrient imbalance, and eutrophication of irrigation rivers and other water bodies [14,15,16]. The deterioration of soil quality and environment rendered changes in fertilizer management; the use of organic fertilizer or green manure to substitute or partially replace chemical fertilizers has been attempted [9,17,18]. However, the complete replacement of chemical fertilizers by organic fertilizer resulted in lower yield [19,20] because the release of plant-available mineral nitrogen from organic sources such as compost or animal manure is slow and often does not keep up with the high crop nitrogen demand during the peak growing period [19,21]. In order to satisfy crop nitrogen demand and achieve high yield, a large amount of manure must be applied, which would leave much nitrogen or subsequent leaching [21].

For small-grain cereals like rice, organic fertilizers are often applied before sowing or seedling transplanting because of their large quantity. The associated disadvantage is the shortage of macronutrients at critical growth stages such as tillering, which adversely affects yield formation. The lower panicle number per unit land area under the OF condition suggested nutrient deficiency at the tillering stage, compared with CF and COF (Table 1). Chemical fertilizers are usually split-applied three or four times during rice growth, achieving high yield by ensuring nutrient supply to plants at all growth stages. Therefore, partial replacement of chemical fertilizer with organic fertilizer has been recommended by researchers: organic fertilizer is used as basal fertilizer, and chemical fertilizer is applied at tillering and panicle initiation [22,23]. In the present study, although the total nitrogen supply in OF was higher than in CF and COF (Table 1), the lower grain yield under OF than under CF or COF treatment confirmed the importance of chemical fertilizer application for achieving high rice yield. The comparable yield under COF and CF conditions suggested that partial replacement of chemical fertilizer with organic fertilizer can maintain yield, meanwhile reducing chemical fertilizer usage; organic fertilizer also improved soil fertility, as shown by the increased mineral nutrient contents in soil (Table S2), which could sustain rice production in the following seasons.

### 3.2. Fertilizer Application on Rice Quality: Organic Fertilizer vs. Chemical Fertilizer

Grain quality, especially the taste of cooked rice, has been claimed to be better for rice grown with organic fertilizer application [24,25]. In the present study, the overall taste under OF was better than under CF and COF, confirming previous claims. The reasons may be associated with differences in grain chalkiness, and amylose and protein content. In the present study, the fertilizer effects on grain quality traits can be classified into two groups: NF and OF vs. COF and CF. Grains harvested under NF and OF conditions had higher grain chalkiness, being whiter and less yellowish in appearance (Table 4). Grains harvested under NF and OF conditions also had lower protein and amino acid concentrations than under COF and CF conditions (Figure 1; Table 7 and Table 8). This difference was mainly attributed to nitrogen fertilization because plants grown under NF and OF conditions did not receive any chemical nitrogen fertilizer after transplanting, whereas plants grown under CF and COF conditions received chemical nitrogen at the tillering and panicle initiation stages (Table S1). Therefore, rice grown under NF and OF conditions may have suffered from nitrogen deficiency during the grain-filling stage, resulting in lower protein and amino acid concentrations in head rice. A review by Bergman and Pandhi [26] on organic rice production practices indicated that conventionally produced rice using chemical fertilizers usually had a greater protein content compared to that produced using an organic method. Organic fertilizers such as compost, green manure, and animal manure provide a slow release of nutrients through mineralization, converting organic nitrogen into ammonium (NH_4_^+^) and nitrate (NO_3_^−^) over time [18]. This explains why although total nitrogen applied under OF treatment was higher than under CF and COF treatments (Table 10), its protein content was lower (Figure 1). Therefore, although viewed as a sustainable approach, organic fertilizers are usually used to complement conventional chemical fertilizers due to their slower nutrient release and labor-intensive management [18].

The differences in rice end-use quality traits between chemical and organic fertilizer practices are associated with grain protein content, which varies with the amount of nitrogen applied during rice growth [26]. Numerous studies have demonstrated that increasing nitrogen supply for rice plants, especially at late growth stages, enhances grain protein content and decreases chalky grain percentage and chalkiness degree [27,28]. In rice endosperm, large amyloplasts or starch granules are embedded among small protein bodies [24,29]; more protein decreases the voids between starch granules, and fewer voids lead to less light diffraction, resulting in a less opaque appearance of rice grains [30,31]. The significant negative correlation between protein concentration and chalky grain percentage (*r* = −0.837 **) in the present study confirmed this assumption.

High contents of protein and amylose reduce the eating quality in *japonica* rice cultivars [28]. Because high protein content affects the water absorption and inhibits the hydration of rice grain during cooking, the viscosity and hardness of the cooked rice change [32]. A recent meta-analysis confirmed that under conventional nitrogen management, increasing the amount of total nitrogen and late-stage nitrogen applications decrease eating quality with significant increases in protein and amylose contents in rice [28]. The application of green manure (Italian ryegrass) and compost (Bokashi) promoted increases in the taste score by reducing amylose and protein contents, in contrast to chemical fertilizer with a higher amount of total nitrogen [24]. Despite the relatively higher total applied nitrogen in OF than CF and COF in the present study, similar results for eating quality-related traits were found: averaged across duckweed treatments, protein (Figure 1) and amylose (Table 5) contents in OF were lower than those in CF and COF; accordingly, the eating quality of cooked rice (Table 6) in OF was better than that in CF and COF. This was largely due to chemical nitrogen application at the late growth stage in CF and COF according to Cheng et al. [28].

### 3.3. Fertilizer-by-Duckweed Interaction on Rice Yield

Duckweed grows naturally in paddy fields, and its coverage during the entire rice cropping season is jointly influenced by fertilizer application and irrigation cycles. Therefore, when interpreting the data, we need to bear in mind that there was no permanent duckweed coverage throughout the rice season in the duckweed treatment under each fertilizer condition. The coverage of duckweed in the paddy field changes over time. After the rice is transplanted, the duckweed is scattered sparsely, and then, it enters a rapid growth period. In plots with ample nutrients (CF, COF, and OF) and during the wet period (with a water layer present) of the dry–wet irrigation cycle, duckweed coverage could reach 100% (visual estimation). In contrast, during the dry period (with no water layer present) of the dry–wet irrigation cycle, duckweed coverage could be close to zero. The average maximum duckweed coverage throughout the rice growth period under NF treatment was approximately 65%, which was generally lower than that in CF, COF, and OF. The present study focused on the cumulative effects of duckweed throughout the entire rice growing season on final yield and quality at harvest.

Previous studies have found that when chemical fertilizers were applied, duckweed coverage in paddy field increased nitrogen use efficiency and grain yield of rice, with yield increases ranging from 4.3% to 15.1% across different studies [8,9]. Recently, a higher yield increase of 16.9% due to duckweed coverage was reported when biogas slurry was used as a sole fertilizer throughout the rice season [10]. In the current study, duckweed coverage during rice growth increased rice yield by 8.3%, averaged across all fertilizer treatments. The magnitude of the yield increase fell within the range reported previously and was solely attributed to increased panicle density (Table 1). Duckweed-induced increases in panicle density were also found in another field study: duckweed growing in paddy fields decreased water temperature and pH, promoted rice growth, and thereby enhanced tiller formation and panicle density [33].

In areas with severe water eutrophication, duckweed outbreaks in irrigation rivers and ditches can cause problems for water management, and duckweed in paddy fields is often viewed as a weed [4]. A recent trial carried out in tanks demonstrated that the duckweed growth–decomposition cycle can be regulated through water management, turning the weed into “fertilizer capacitors” [34]. In the present study, wet–dry alternation irrigation was employed throughout the entire rice season. The frequent irrigation–drainage cycles promoted the duckweed growth–decomposition cycles, thereby enabling continuous capture and release of nutrients. Nitrogen is a key factor for tiller formation, especially in fields with heavy straw-returning practices, where straw-decomposing microbes compete with rice for nitrogen [35]. In addition, this irrigation method can control duckweed growth, avoiding overgrowth that might otherwise create an unfavorable environment for rice tiller formation.

### 3.4. Fertilizer-by-Duckweed Interaction on Rice Quality

Significant fertilizer-by-duckweed interactions were detected in grain chalkiness, protein or amino acid concentrations, palatability of cooked rice, and most parameters of the RVA profile. These interactions were mainly caused by the distinct duckweed effect under the NF condition, which was opposite to the effects under CF, COF, and OF conditions. Duckweed coverage under the NF condition decreased the concentrations of protein, essential, and non-essential amino acids in milled rice (Figure 1 and Table 7), probably because without fertilizer supply, duckweed competed with rice for nitrogen and other mineral elements essential for amino acids and protein synthesis. In contrast, with fertilizer supply, such competition was less intense, and duckweed may even increase rice nitrogen use efficiency by preventing NH_3_ loss, and promote rice nutrient uptake through duckweed decomposition and nutrient release during light dry–wet irrigation cycles [7].

Duckweed’s effect on rice nutrient uptake, especially nitrogen uptake, under different fertilizer conditions resulted in different protein changes in grains. Previous studies have demonstrated that the protein content in rice grains is closely related to grain chalkiness [28] and cooking and eating quality [32]. In the present study, duckweed coverage under NF decreased protein concentration (Figure 1) and the setback value of the RVA profile (Table 5) but increased chalky grain percentage (Table 4) and overall palatability index (Table 6). On the contrary, duckweed coverage tended to increase protein concentration and the setback value of RVA profile but decrease chalky grain percentage and overall palatability index under conditions of fertilizer application. These results are consistent with previous studies on the relationship between grain protein concentration and grain chalkiness/cooking and eating quality traits, suggesting that protein changes induced by duckweed or fertilizer were one reason for the observed changes in grain appearance and palatability.

Among all quality traits measured in this experiment, the most prominent duckweed effect on rice quality was reduced zinc concentration in grains across all four fertilizer treatments (Table 9). Grain zinc reduction was also detected in our preliminary study conducted in fertile lotus-pond bottom soil [11]. The present study, conducted in an open paddy field, confirmed that duckweed growing in paddies decreases the zinc nutrition of rice. Duckweed can take up and accumulate large amounts of zinc [36,37,38], thereby competing with rice for zinc ions, especially given that soil available zinc content in this location was low, only 1.4 mg kg^−1^ under NF and 1.5 mg kg^−1^ under CF (Table S2). Interestingly, the soil available zinc content was 6.3 mg kg^−1^ under COF and 18.1 mg kg^−1^ under OF, much higher than that under CF and NF conditions, although zinc concentration variations in rice grains between fertilizer treatments were relatively smaller (Table 9). The high soil available zinc content in COF and OF plots was due to the application of organic fertilizer, confirming soil micronutrient enrichment by organic fertilizers.

### 3.5. Study Limitations

While this study provides valuable insights into duckweed–fertilizer interactions on rice production, several limitations should be acknowledged. First, the results are based on a single-year experiment, which did not take into account the interannual variations in meteorological conditions that could impact duckweed growth. Previous studies have demonstrated that duckweed growth is sensitive to temperature and light intensity [1,39]. Thus, fluctuations in these environmental factors across years could potentially modify duckweed’s effects on rice yield formation and grain quality traits. Second, the experiment was conducted at a single location with a specific irrigation practice. The observed interactions between duckweed and fertilizers might vary in other locations with different water management regimes. Third, the experiment used one *japonica* cultivar; whether similar findings occur in other *japonica* cultivars or *indica* cultivars remains unclear. Further trials with multi-year, multi-location, and multi-cultivar designs are needed to verify the findings.

## 4. Materials and Methods

### 4.1. Experimental Site and Design

The experimental site was located at the Modern Agricultural Experimental Demonstration Base of Xuzhou Academy of Agricultural Sciences in Xuzhou City, Jiangsu Province. This area has a temperate monsoon climate. The average annual temperature is 14 °C, and the average annual precipitation ranges from 800 to 930 mm. The actual weather conditions (daily air temperature and precipitation) of the experimental site during the rice growing season are shown in Figure S2. The soil has a sandy loam texture, with a sand content of 58.0% sand, silt 29.4%, and clay 12.6%. The experiments were conducted with a split-plot design in three replications and eight treatments. The main factor was fertilizer options: (1) organic fertilizer (OF); (2) chemical fertilizer (CF); (3) mix (one-third of OF and two-thirds of CF based on nitrogen content, COF); and (4) no fertilizer (NF), which was also simultaneously evaluated to assess background contributions from the soil. The sub-factor was duckweed treatments: no duckweed coverage (Control) and duckweed coverage (Duckweed). The size of each fertilizer plot was 200 m^2^.

### 4.2. Crop Cultivation

The seeds of *japonica* rice cultivar Xudao 9 were sown in seedling trays on 25 May 2023. Uniform seedlings were manually transplanted to experimental plots on June 30, with 3 rice seedlings per hill and a transplanting density of 13.3 cm by 30 cm. On 15 July 2023, when duckweed started emerging naturally, each fertilizer treatment plot was divided into two sub-plots of a no-duckweed (control) area and a duckweed-covered area by inserting a plastic board. The no-duckweed area was maintained by regularly removing duckweed with a mesh net.

The water management method deployed in the experimental field was light wet–dry alternate irrigation: transplanting was performed with a shallow water level of around 3 cm, and this shallow water layer was maintained for 3 days after transplanting. The water level then decreased naturally through evapotranspiration and percolation until the water layer disappears (though small puddles remained visible). The duration of this process depends on the weather, typically 2–3 days on sunny days. The field was kept free of a water layer for another 2–3 days; by this point, most small puddles had disappeared, and the soil moisture content was approximately 100%. Watering was then restarted. This wet–dry cycle was maintained throughout the rice season except in late July, when the field was subjected to continuous drainage until the tillage layer hardened to prevent excess tiller formation. Irrigation was completely stopped one week before harvest. Pests and diseases were controlled following local farmers’ practices.

### 4.3. Fertilizer Application

Before rice transplanting, basal fertilizer was applied to the experimental plots. The organic fertilizer (Kuaibang, Xuzhou Kuaibang Biotechnology Development Co., Ltd., Xuzhou, China), for which its major raw material is cow manure, was used solely as a basal fertilizer. The contents of organic matter (determined by potassium dichromate oxidation), total nitrogen (determined by Kjeldahl method), phosphorus (P_2_O_5_, determined by H_2_SO_4_–HClO_4_ digestion and ammonium molybdate spectrophotometry), and potassium (K_2_O, determined by HNO_3_–HClO_4_–HF digestion and flame photometry) in the organic fertilizer, measured in our laboratory, were 347 g kg^−1^, 12.2 g kg^−1^, 7.4 g kg^−1^, and 10.2 g kg^−1^, respectively. All organic fertilizer for the OF and COF treatments was applied as basal fertilizer. For the CF and COF treatments, compound chemical fertilizer with a N:P_2_O_5_:K_2_O ratio of 15:15:15 was applied before transplanting as basal fertilizer and at the rice tillering stage; urea was split-applied before transplanting, and at the tillering and panicle initiation stages for the CF and COF treatments. Detailed information on fertilizer application is shown in Table 10 and Table S1.

### 4.4. Sampling and Parameter Measurements

Plants were harvested on 12 October 2023. After harvest, soil samples (0–15 cm depth) from each plot were collected; processed; and analyzed for pH, total N, total P, alkali-hydrolysable N, Olsen-P, exchangeable K, available Fe, available Mn, available Cu, and available Zn. The soil analysis followed the methods described by Mi et al. [40].

For theoretical yield determination, the average tiller number from 50 plants per plot (a patch of 2 m^2^ land area) were recorded prior to sampling. Three representative hills with tiller numbers close to the plot average were then selected to evaluate grain yield and yield components (panicle number, spikelet number per panicle, filled-grain percentage, and individual grain weight), based on a previously reported method [11].

For rice quality measurements, grains were dehulled to obtain brown rice, which was then polished by a rice milling machine (LTJM-2099, Zhejiang Top Instrument Co., Ltd., Hangzhou, China) for 25 s to produce milled rice. Head rice was manually picked up following the criteria set by the Chinese national standard: the milled rice with a length greater than or equal to three-quarters of the whole kernel. The brown rice percentage, milled rice percentage, or head rice percentage are expressed as the weight percentage of brown rice, milled rice, or head rice obtained from a sample of rough rice.

The appearance quality of head rice, including grain shape, chalky-grain percentage, chalkiness degree, Ym, and white degree, were determined by a rice appearance quality scanner equipped with software. (JMWT12, Beijing Dongfu Jiuheng Instrument Technology Co. Ltd., Beijing, China) Then, the amylose and protein contents of head rice were determined by the Infratec 1241 Grain Quality Analyzer (FOSS Tecator, Hillerod, Denmark). After that, 10 g of head rice was cooked in a rice cooker and immediately subjected to taste assessment using a rice taste analyzer (STA1A, SA-TAKE Co., Ltd., Hiroshima, Japan).

Another 20 g of head rice was ground to powder for the following analysis of cooking quality and nutritional quality. The starch pasting property of rice powder was assessed by RVA according to the protocol from the American Association of Cereal Chemists (AACC 1995-61-02). Determination of amino acid concentrations in polished rice followed the methods by Zhou et al. [41]. In brief, rice powder was digested by HCl. After a series of purification, the samples were subjected to HPLC (Waters 2695, Milford, MA, USA) for the determination of amino acid concentrations.

Mineral element content in milled rice was measured as follows: 0.45 g of rice powder was digested by concentrated nitric acid in a microwave digestion apparatus (MARS5, CEM Corporation, Matthews, NC, USA). After dilution and filtration, the concentrations of elements in the filtrate were determined by ICP-AES (iCAP6300, Thermo Fisher Scientific, Waltham, MA, USA). Quality control was performed using a reference material (GBW08503b, National Institute of Metrology, Beijing, China).

### 4.5. Statistical Analysis

All data were analyzed using SPSS statistical software (SPSS, 26.0, IBM Corporation, Chicago, IL, USA). Univariate analysis of variance (ANOVA) and interactions between fertilizer application and duckweed treatments were carried out, using fertilizer and duckweed treatment as fixed factors and block as random factor. Pearson correlation analysis was conducted to evaluate relationships between quality traits. Data in tables and graphs represent means with standard errors of three replicates. ** indicates *p* < 0.01, and * indicates *p* < 0.05.

## 5. Conclusions

Our results suggest that duckweed’s effect on rice yield and quality may vary with fertilizer applications in paddy fields. Although duckweed generally had a positive effect on rice yield, its effect on grain quality was complex, showing both positive and negative outcomes depending on specific traits and fertilizer regimes. The decrease in grain copper and zinc concentration may impose further “hidden hunger”, suggesting micronutrient fertilizers may be required in rice production where duckweed occurrence is common. Partial replacement of chemical fertilizers with organic fertilizers is a promising cultivation strategy in paddy fields, as it better balances rice yield and quality. However, these findings are based on a single growing season under specific experimental conditions, and further multi-year studies across diverse environmental conditions are needed to validate these trends. Future research should further explore integrated duckweed and fertilizer management strategies to mitigate potential negative impacts while enhancing the positive effects of duckweed on rice production.

## Figures and Tables

**Table 1:** Effects of duckweed coverage on grain yield and yield components under different fertilization conditions.

Fertilizer Treatment	Duckweed Treatment	Panicle Number (m^−2^)	Spikelet Number per Panicle	Filled-Grain Percentage (%)	Individual Grain Weight (mg)	Grain Yield (kg ha^−1^)
NF	Control	116.9 ± 0.0	173.6 ± 9.5	92.6 ± 2.4	26.1 ± 0.1	4864 ± 187.1
	Duckweed	142.0 ± 0.0 **	170.4 ± 6.1	93.6 ± 1.4	26.8 ± 0.8	6033 ± 192.5 *
CF	Control	328.6 ± 2.8	182.6 ± 12.9	81.2 ± 2.2	24.7 ± 0.4	11,994 ± 337.7
	Duckweed	325.8 ± 0.0	175.3 ± 5.7	83.8 ± 1.6	25.0 ± 0.4	11,945 ± 391.3
COF	Control	298.0 ± 2.8	190.3 ± 6.9	77.4 ± 4.6	25.0 ± 0.2	9358 ± 403.1
	Duckweed	375.9 ± 0.0 **	172.6 ± 8.6	77.1 ± 3.1	24.5 ± 0.4	10,129 ± 399.8
OF	Control	192.2 ± 0.0	189.4 ± 9.8	94.8 ± 0.5	27.3 ± 0.3	10,920 ± 407.9
	Duckweed	256.2 ± 2.8 **	186.3 ± 17.1	83.1 ± 3.0 *	26.1 ± 0.9	12,129 ± 429.2
ANOVA results						
Fertilizer		**<0.001**	0.502	**<0.001**	**0.002**	**<0.001**
Duckweed		**<0.001**	0.295	0.283	0.533	**0.007**
Fertilizer × Duckweed		**<0.001**	0.876	0.057	0.275	0.295

NF, no fertilizer application; CF, chemical fertilizer; COF, two-thirds of CF and one-third of organic fertilizer; OF, organic fertilizer. Control, no duckweed coverage; Duckweed, duckweed coverage. Values are means ± standard errors. ** or * indicates significant duckweed effect at *p* < 0.01 or *p* < 0.05, respectively. ANOVA values in bold indicate significance at *p* < 0.05.

**Table 2:** Effects of duckweed coverage on grain processing quality under different fertilization conditions.

Fertilizer Treatment	Duckweed Treatment	Brown Rice Percentage (%)	Milled Rice Percentage (%)	Head Rice Percentage (%)
NF	Control	82.8 ± 0.2	73.1 ± 0.2	65.4 ± 0.6
	Duckweed	82.8 ± 0.3	73.1 ± 0.4	69.3 ± 1.0
CF	Control	83.6 ± 0.5	72.7 ± 1.1	68.8 ± 0.8
	Duckweed	83.0 ± 0.7	71.9 ± 1.0	68.2 ± 1.6
COF	Control	83.1 ± 0.3	71.5 ± 0.5	67.7 ± 0.6
	Duckweed	82.6 ± 0.7	71.3 ± 1.3	67.1 ± 1.3
OF	Control	83.8 ± 0.1	74.1 ± 0.2	69.6 ± 0.8
	Duckweed	84.0 ± 0.1	73.5 ± 0.1 *	68.7 ± 1.2
ANOVA results				
Fertilizer		0.132	**0.039**	0.465
Duckweed		0.130	0.074	0.414
Fertilizer × Duckweed		0.339	0.501	**0.027**

NF, no fertilizer application; CF, chemical fertilizer; COF, two-thirds of CF and one-third of organic fertilizer; OF, organic fertilizer. Control, no duckweed coverage; Duckweed, duckweed coverage. Values are means ± standard errors. * indicates significant duckweed effect at *p* < 0.05. ANOVA values in bold indicate significance at *p* < 0.05.

**Table 3:** Effects of duckweed coverage on appearance quality of brown rice under different fertilization conditions.

Fertilizer Treatment	Duckweed Treatment	Weight Ratio of Immature Grain (%)	Length (mm)	Width (mm)	Length–Width Ratio (L/W)	Ym	White Degree
NF	Control	6.5 ± 0.5	5.26 ± 0.01	2.65 ± 0.02	1.98 ± 0.02	48.2 ± 0.6	16.0 ± 0.4
	Duckweed	7.2 ± 0.3	5.28 ± 0.01 *	2.62 ± 0.00	2.02 ± 0.01	48.4 ± 0.6	16.1 ± 0.3
CF	Control	8.1 ± 1.5	5.32 ± 0.01	2.71 ± 0.02	1.97 ± 0.01	49.9 ± 0.5	15.0 ± 0.2
	Duckweed	10.0 ± 1.2	5.39 ± 0.02 *	2.72 ± 0.01	1.98 ± 0.00	51.0 ± 0.6	14.5 ± 0.3
COF	Control	11.1 ± 0.4	5.36 ± 0.03	2.72 ± 0.01	1.97 ± 0.00	51.4 ± 0.6	14.0 ± 0.3
	Duckweed	10.7 ± 1.4	5.45 ± 0.04	2.76 ± 0.02	1.98 ± 0.01	51.3 ± 0.2	14.3 ± 0.1
OF	Control	5.0 ± 0.4	5.38 ± 0.02	2.72 ± 0.01	1.98 ± 0.00	48.0 ± 0.1	16.3 ± 0.0
	Duckweed	8.1 ± 0.4 **	5.41 ± 0.01 *	2.74 ± 0.01	1.98 ± 0.00	48.5 ± 0.3	16.1 ± 0.2
ANOVA results							
Fertilizer		**0.023**	**0.012**	**0.002**	**0.025**	**0.007**	**0.003**
Duckweed		**0.023**	**<0.001**	0.106	**0.031**	0.072	0.347
Fertilizer × Duckweed		0.132	**0.047**	**0.009**	0.164	0.227	0.161

NF, no fertilizer application; CF, chemical fertilizer; COF, two-thirds of CF and one-third of organic fertilizer; OF, organic fertilizer. Control, no duckweed coverage; Duckweed, duckweed coverage. Ym, the yellow index of rice grains. Values are means ± standard errors. ** or * indicates significant duckweed effect at *p* < 0.01 or *p* < 0.05, respectively. ANOVA values in bold indicate significance at *p* < 0.05.

**Table 4:** Effects of duckweed coverage on grain shape, chalky-grain percentage, chalkiness degree, Ym, and white degree of head rice under different fertilization conditions.

Fertilizer Treatment	Duckweed Treatment	Length (mm)	Width (mm)	Length–Width Ratio (L/W)	Chalky-Grain Percentage (%)	Chalkiness Degree (%)	Ym	White Degree
NF	Control	4.86 ± 0.02	2.59 ± 0.02	1.87 ± 0.01	67.9 ± 1.2	25.1 ± 1.7	15.9 ± 0.1	39.5 ± 0.1
	Duckweed	4.85 ± 0.02	2.56 ± 0.01	1.90 ± 0.01	79.4 ± 1.8 *	32.6 ± 1.9	16.0 ± 0.1	40.0 ± 0.3
CF	Control	4.93 ± 0.01	2.63 ± 0.01	1.87 ± 0.00	44.9 ± 5.8	13.8 ± 2.3	17.8 ± 0.3	37.2 ± 0.4
	Duckweed	5.00 ± 0.02 *	2.67 ± 0.01 **	1.87 ± 0.01	29.8 ± 7.5 *	9.2 ± 2.6 *	19.4 ± 0.9	35.5 ± 1.1
COF	Control	4.95 ± 0.01	2.66 ± 0.01	1.87 ± 0.01	32.7 ± 11.7	10.1 ± 3.6	18.9 ± 1.1	36.0 ± 1.3
	Duckweed	5.01 ± 0.03	2.67 ± 0.02	1.88 ± 0.01	24.0 ± 5.5	7.9 ± 1.7	20.0 ± 0.9 *	35.0 ± 1.0
OF	Control	4.98 ± 0.02	2.64 ± 0.01	1.88 ± 0.00	68.8 ± 2.1	22.6 ± 0.8	16.6 ± 0.2	38.8 ± 0.2
	Duckweed	5.00 ± 0.02	2.66 ± 0.01	1.88 ± 0.01	63.9 ± 3.4	21.6 ± 1.7	16.6 ± 0.3	38.9 ± 0.3
ANOVA results								
Fertilizer		**0.004**	**0.001**	0.256	**0.003**	**0.001**	**0.014**	**0.009**
Duckweed		**0.018**	**0.041**	0.244	0.071	0.925	**0.006**	**0.023**
Fertilizer × Duckweed		0.152	**0.011**	0.179	**0.010**	**0.007**	**0.042**	**0.022**

NF, no fertilizer application; CF, chemical fertilizer; COF, two-thirds of CF and one-third of organic fertilizer; OF, organic fertilizer. Control, no duckweed coverage; Duckweed, duckweed coverage. Ym, the yellow index of rice grains. Values are means ± standard errors. ** or * indicates significant duckweed effect at *p* < 0.01 or *p* < 0.05, respectively. ANOVA values in bold indicate significance at *p* < 0.05.

**Table 5:** Effects of duckweed coverage on amylose content and RVA profiles of milled rice flours under different fertilization conditions.

Fertilizer Treatment	Duckweed Treatment	Amylose Content (%)	Peak Viscosity (cP)	Hot Viscosity (cP)	Breakdown (cP)	Final Viscosity (cP)	Setback (cP)	Peak Time (min)	Pasting Temperature (°C)
NF	Control	11.6 ± 0.8	4675 ± 38	2283 ± 32	2393 ± 42	3306 ± 20	−1369 ± 18	5.78 ± 0.02	71.98 ± 0.46
	Duckweed	10.5 ± 0.2	4813 ± 130	2144 ± 138	2669 ± 69	3038 ± 117	−1775 ± 98 *	5.69 ± 0.04 *	72.03 ± 0.02
CF	Control	13.4 ± 0.3	4347 ± 49	1957 ± 27	2390 ± 62	2876 ± 29	−1471 ± 48	5.71 ± 0.02	70.97 ± 0.24
	Duckweed	13.2 ± 0.3	4250 ± 161	2147 ± 131	2104 ± 31 *	3068 ± 121	−1183 ± 44 **	5.89 ± 0.02 *	71.97 ± 0.43
COF	Control	13.0 ± 0.4	4322 ± 146	2072 ± 86	2249 ± 62	2970 ± 113	−1352 ± 62	5.75 ± 0.02	70.93 ± 0.72
	Duckweed	13.4 ± 0.4	4058 ± 207	1827 ± 144	2231 ± 63	2670 ± 186	−1388 ± 24	5.62 ± 0.05	70.18 ± 0.94
OF	Control	12.1 ± 0.2	4482 ± 203	2034 ± 122	2449 ± 122	2963 ± 136	−1519 ± 130	5.71 ± 0.06	70.68 ± 0.54
	Duckweed	13.2 ± 0.4	4544 ± 92	2225 ± 103 *	2320 ± 12	3082 ± 106	−1463 ± 16	5.73 ± 0.04	71.52 ± 0.51 **
ANOVA results									
Fertilizer		**0.014**	**0.019**	**0.043**	**0.017**	**0.032**	0.061	**0.015**	0.322
Duckweed		0.880	0.551	0.989	0.393	0.248	0.596	0.887	0.127
Fertilizer × Duckweed		0.077	0.208	0.071	**0.011**	**0.019**	**0.004**	**0.019**	**0.021**

NF, no fertilizer application; CF, chemical fertilizer; COF, two-thirds of CF and one-third of organic fertilizer; OF, organic fertilizer. Control, no duckweed coverage; Duckweed, duckweed coverage. Values are means ± standard errors. ** or * indicates significant duckweed effect at *p* < 0.01 or *p* < 0.05, respectively. ANOVA values in bold indicate significance at *p* < 0.05.

**Table 6:** Effects of duckweed coverage on the palatability of cooked rice under different fertilization conditions.

Fertilizer Treatment	Duckweed Treatment	Overall Palatability Index	Luster	Hardness	Stickiness	Balance Degree
NF	Control	69.3 ± 3.8	6.6 ± 0.6	6.4 ± 0.4	6.4 ± 0.5	6.5 ± 0.6
	Duckweed	82.0 ± 3.1 *	8.4 ± 0.4 *	5.4 ± 0.3 *	8.2 ± 0.3 *	8.4 ± 0.4 *
CF	Control	71.0 ± 2.5	6.8 ± 0.4	6.2 ± 0.2	6.5 ± 0.4	6.8 ± 0.4
	Duckweed	63.0 ± 2.0 **	5.5 ± 0.3 **	6.9 ± 0.1 *	5.5 ± 0.5	5.5 ± 0.3 **
COF	Control	61.7 ± 2.6	5.4 ± 0.4	6.9 ± 0.1	5.3 ± 0.5	5.3 ± 0.4
	Duckweed	70.0 ± 0.6	6.7 ± 0.1	6.3 ± 0.1	6.3 ± 0.2	6.6 ± 0.2
OF	Control	75.3 ± 2.3	7.5 ± 0.4	5.9 ± 0.2	7.2 ± 0.4	7.4 ± 0.4
	Duckweed	75.0 ± 1.5	7.5 ± 0.3	5.9 ± 0.2	7.2 ± 0.3	7.4 ± 0.3
ANOVA results						
Fertilizer		**0.034**	**0.033**	0.068	**0.025**	**0.033**
Duckweed		**0.035**	0.055	**0.045**	0.096	0.064
Fertilizer × Duckweed		**0.002**	**0.002**	**0.001**	**0.021**	**0.003**

NF, no fertilizer application; CF, chemical fertilizer; COF, two-thirds of CF and one-third of organic fertilizer; OF, organic fertilizer. Control, no duckweed coverage; Duckweed, duckweed coverage. Values are means ± standard errors. ** or * indicates significant duckweed effect at *p* < 0.01 or *p* < 0.05, respectively. ANOVA values in bold indicate significance at *p* < 0.05.

**Table 7:** Effects of duckweed coverage on essential, non-essential, and total amino acid concentrations (g kg^−1^) in milled rice under different fertilization conditions.

Fertilizer Treatment	Duckweed Treatment	Essential Amino Acid	Non-Essential Amino Acid	Total Amino Acid
NF	Control	20.42 ± 1.32	38.72 ± 2.56	59.14 ± 3.87
	Duckweed	18.11 ± 0.92 *	34.10 ± 1.99 *	52.21 ± 2.91 *
CF	Control	22.86 ± 0.94	41.00 ± 0.94	63.86 ± 1.64
	Duckweed	23.14 ± 0.64	44.29 ± 1.23	67.43 ± 1.87
COF	Control	22.29 ± 1.18	41.50 ± 1.87	63.78 ± 3.01
	Duckweed	21.63 ± 0.11	41.74 ± 0.38	63.37 ± 0.48
OF	Control	19.86 ± 0.21	37.70 ± 0.33	57.56 ± 0.54
	Duckweed	21.42 ± 0.86	39.20 ± 0.66	60.62 ± 1.33
ANOVA results				
Fertilizer		0.054	**0.032**	**0.036**
Duckweed		0.597	0.902	0.891
Fertilizer × Duckweed		0.123	**0.040**	0.060

NF, no fertilizer application; CF, chemical fertilizer; COF, two-thirds of CF and one-third of organic fertilizer; OF, organic fertilizer. Control, no duckweed coverage; Duckweed, duckweed coverage. Values are means ± standard errors. * indicates significant duckweed effect at *p* < 0.05. ANOVA values in bold indicate significance at *p* < 0.05.

**Table 8:** Effects of duckweed coverage on the concentrations of essential and non-essential amino acids (g kg^−1^) in milled rice under different fertilization conditions.

Fertilizer Treatment	Duckweed Treatment	Essential Amino Acid								
		Thr		Val	Met	Ile	Leu	Phe	Lys	
NF	Control	2.15 ± 0.13		3.85 ± 0.25	1.12 ± 0.07	2.55 ± 0.19	5.28 ± 0.37	3.27 ± 0.20	2.20 ± 0.11	
	Duckweed	1.91 ± 0.10 *		3.39 ± 0.17 *	0.97 ± 0.03	2.26 ± 0.12	4.67 ± 0.25	2.87 ± 0.19 *	2.03 ± 0.07 *	
CF	Control	2.28 ± 0.05		4.55 ± 0.31	1.67 ± 0.26	2.93 ± 0.14	5.67 ± 0.15	3.48 ± 0.16	2.27 ± 0.03	
	Duckweed	2.40 ± 0.06		4.31 ± 0.09	1.15 ± 0.07	2.94 ± 0.07	6.06 ± 0.13	3.85 ± 0.13	2.44 ± 0.10	
COF	Control	2.29 ± 0.09		4.33 ± 0.35	1.31 ± 0.20	2.79 ± 0.15	5.69 ± 0.23	3.65 ± 0.15	2.23 ± 0.06	
	Duckweed	2.21 ± 0.06		4.06 ± 0.04	1.12 ± 0.03	2.73 ± 0.00	5.69 ± 0.04	3.54 ± 0.03	2.28 ± 0.04	
OF	Control	2.07 ± 0.01		3.71 ± 0.05	1.05 ± 0.03	2.52 ± 0.03	5.12 ± 0.05	3.30 ± 0.05	2.09 ± 0.03	
	Duckweed	2.13 ± 0.03		4.15 ± 0.29	1.34 ± 0.29	2.72 ± 0.14	5.45 ± 0.12 *	3.41 ± 0.06	2.23 ± 0.06	
ANOVA results										
Fertilizer		**0.030**		0.103	0.308	0.055	**0.038**	**0.019**	0.146	
Duckweed		0.422		0.366	0.245	0.633	0.788	0.910	0.196	
Fertilizer × Duckweed		0.071		0.196	0.127	0.189	**0.027**	0.065	**0.016**	
Fertilizer Treatment	Duckweed Treatment	Non-essential amino acid								
		Asp	Ser	Glu	Gly	Ala	Tyr	His	Arg	Pro
NF	Control	6.34 ± 0.35	3.08 ± 0.22	11.6 ± 0.85	2.96 ± 0.16	3.69 ± 0.17	2.15 ± 0.24	1.31 ± 0.10	5.34 ± 0.36	2.23 ± 0.14
	Duckweed	5.70 ± 0.42 *	2.71 ± 0.13	10.1 ± 0.61 *	2.68 ± 0.12 *	3.24 ± 0.14 **	1.81 ± 0.14	1.14 ± 0.07 *	4.73 ± 0.26 *	1.99 ± 0.16 *
CF	Control	6.86 ± 0.10	3.23 ± 0.10	12.1 ± 0.27	3.06 ± 0.06	3.80 ± 0.05	2.42 ± 0.11	1.35 ± 0.05	5.66 ± 0.17	2.50 ± 0.16
	Duckweed	7.22 ± 0.11	3.49 ± 0.08	13.2 ± 0.32	3.29 ± 0.08	4.09 ± 0.12	2.56 ± 0.25	1.49 ± 0.04	6.15 ± 0.22	2.83 ± 0.07
COF	Control	6.87 ± 0.32	3.33 ± 0.14	12.4 ± 0.51	3.16 ± 0.12	3.93 ± 0.13	2.31 ± 0.18	1.39 ± 0.07	5.74 ± 0.27	2.34 ± 0.14
	Duckweed	7.25 ± 0.41	3.01 ± 0.29	12.4 ± 0.06	3.12 ± 0.01	3.91 ± 0.03	2.42 ± 0.11	1.38 ± 0.02	5.81 ± 0.08	2.46 ± 0.01
OF	Control	6.01 ± 0.04	3.00 ± 0.03	11.2 ± 0.09	2.87 ± 0.03	3.54 ± 0.02	2.25 ± 0.06	1.25 ± 0.01	5.27 ± 0.06	2.37 ± 0.07
	Duckweed	6.31 ± 0.11	3.14 ± 0.04	11.7 ± 0.13 *	2.99 ± 0.05	3.70 ± 0.02 *	2.22 ± 0.12	1.32 ± 0.03	5.44 ± 0.10	2.43 ± 0.19
ANOVA results										
Fertilizer		**0.048**	0.060	**0.044**	**0.044**	**0.028**	**0.040**	0.060	**0.037**	**0.035**
Duckweed		0.502	0.494	0.988	0.856	0.954	0.811	0.825	0.789	0.426
Fertilizer × Duckweed		0.091	0.152	**0.015**	**0.027**	**0.008**	0.556	**0.025**	0.082	0.144

NF, no fertilizer application; CF, chemical fertilizer; COF, two-thirds of CF and one-third of organic fertilizer; OF, organic fertilizer. Control, no duckweed coverage; Duckweed, duckweed coverage. Thr, threonine; Val, valine; Met, methionine; Ile, isoleucine; Leu, leucine; Phe, phenylalanine; Lys, lysine; Asp, aspartate; Ser, serine; Glu, glutamate; Gly, glycine; Ala, alanine; Tyr, tyrosine; His, histidine; Arg, arginine; Pro, proline. Values are means ± standard errors. ** or * indicates significant duckweed effect at *p* < 0.01 or *p* < 0.05, respectively. ANOVA values in bold indicate significance at *p* < 0.05.

**Table 9:** Effects of duckweed coverage on the element concentrations of milled rice under different fertilization conditions.

Fertilizer Treatment	Duckweed Treatment	Macro-Element Concentration				Micro-Element Concentration			
		Ca (g kg^−1^)	K (g kg^−1^)	Mg (g kg^−1^)	*p* (g kg^−1^)	Fe (mg kg^−1^)	Mn (mg kg^−1^)	Cu (mg kg^−1^)	Zn (mg kg^−1^)
NF	Control	0.10 ± 0.01	0.48 ± 0.01	0.18 ± 0.00	1.06 ± 0.01	19.11 ± 2.04	6.64 ± 0.17	3.61 ± 0.94	11.57 ± 0.43
	Duckweed	0.09 ± 0.00	0.50 ± 0.01 *	0.18 ± 0.00	1.14 ± 0.04	20.15 ± 3.68	6.40 ± 0.02	2.13 ± 0.05	9.14 ± 0.16 *
CF	Control	0.09 ± 0.00	0.59 ± 0.01	0.14 ± 0.00	1.10 ± 0.04	21.13 ± 1.29	7.52 ± 0.35	2.57 ± 0.18	8.11 ± 0.57
	Duckweed	0.09 ± 0.00	0.66 ± 0.06	0.14 ± 0.02	0.95 ± 0.07	21.33 ± 3.94	7.79 ± 0.62	1.99 ± 0.16 *	6.98 ± 0.33 *
COF	Control	0.09 ± 0.01	0.64 ± 0.05	0.13 ± 0.01	0.80 ± 0.06	17.46 ± 1.99	7.21 ± 0.33	2.85 ± 0.46	8.68 ± 0.60
	Duckweed	0.09 ± 0.01	0.66 ± 0.03	0.12 ± 0.01	0.84 ± 0.12	18.04 ± 3.32	6.70 ± 0.54	1.99 ± 0.30 *	7.38 ± 0.68 *
OF	Control	0.08 ± 0.00	0.50 ± 0.00	0.16 ± 0.00	1.01 ± 0.13	22.79 ± 5.49	6.35 ± 0.08	2.20 ± 0.22	9.41 ± 0.26
	Duckweed	0.09 ± 0.00	0.52 ± 0.00 *	0.15 ± 0.00 *	0.87 ± 0.10	27.39 ± 10.16	6.40 ± 0.05	1.74 ± 0.17 *	8.16 ± 0.27 *
ANOVA results									
Fertilizer		0.264	**0.012**	**0.004**	0.063	0.400	0.151	0.158	**0.007**
Duckweed		0.894	0.054	0.205	0.190	0.505	0.463	**0.014**	**<0.001**
Fertilizer × Duckweed		0.325	0.552	0.757	**0.039**	0.898	0.293	0.451	**0.039**

NF, no fertilizer application; CF, chemical fertilizer; COF, two-thirds of CF and one-third of organic fertilizer; OF, organic fertilizer. Control, no duckweed coverage; Duckweed, duckweed coverage. Ca, calcium; K, potassium; Mg, magnesium; P, phosphorus; Fe, iron; Mn, manganese; Cu, copper; Zn, zinc. Values are means ± standard errors. * indicates significant duckweed effect at *p* < 0.05. ANOVA values in bold indicate significance at *p* < 0.05.

**Table 10:** Amount of N, P_2_O_5_, and K_2_O (g m^−2^) applied in treatments from different organic and chemical fertilizers sources.

Fertilizer Treatments	Organic Fertilizer	Chemical Fertilizer	Total
No fertilizer (NF)	0:0:0	0:0:0	0:0:0
Chemical fertilizer (CF)	0:0:0	29:10:10	29:10:10
Mix (two-thirds of CF and one-third of organic fertilizer, COF)	12.2:7.4:10.2	19.3:6.8:6.8	31.5:14.2:17.0
Organic fertilizer (OF)	36.6:22.2:30.6	0:0:0	36.6:22.2:30.6

Fertilizer application rates are shown in the form of N:P_2_O_5_:K_2_O.

## Data Availability

The original data contributions presented in this study are included in the article/Supplementary Materials. Further inquiries can be directed to the Corresponding Authors.

## Supplementary Materials

The following supporting information can be downloaded at https://www.mdpi.com/article/10.3390/plants14182850/s1, Figure S1: Effects of duckweed coverage on the RVA profiles of milled rice flours under different fertilization conditions; Figure S2: Daily air temperature and precipitation at the experiment site during rice growing season (from transplanting to harvest); Table S1: The fertilizer application scheme in the experiment; Table S2: Effect of duckweed coverage on the soil properties under different fertilization conditions.

## Abbreviations

The following abbreviations are used in this manuscript:

NF	no fertilizer application
CF	chemical fertilizer
COF	two-thirds of the chemical fertilizer and one-third of the organic fertilizer
OF	organic fertilizer
