# Supplementary material for: Duckweed’s Effects on Rice Yield and Quality Varied with Fertilizer Applications
Source: Plants (Basel). 2025 Sep 12;14(18):2850. doi: 10.3390/plants14182850 (PMC12473964; doi:10.3390/plants14182850)
Supplement: Supplementary file 1 [file plants-14-02850-s001.zip › plants-3628278-supplementary.pdf]

Supplementary Materials

Table S1. The fertilizer application scheme in the experiment.

Fertilizer Treatment	Basal Application (kg ha^{-1})			Tillering (kg ha^{-1})		Panicle Initiation (kg ha^{-1})
	Organic Fertilizer	Compound Chemical Fertilizer	Urea	Compound Chemical Fertilizer	Urea	Urea
NF	0	0	0	0	0	0
CF	0	450	150	225	150	112.5
COF	10000	300	100.5	150	100.5	75
OF	30000	0	0	0	0	0

NF, no fertilizer application; CF, chemical fertilizer; COF, two-third of CF and one-third of organic fertilizer; OF, organic fertilizer.

Table S2. Effect of duckweed coverage on the soil properties under different fertilization conditions.

Fertilizer Treatment	Duckweed Treatment	pH	Total N (g kg^{-1})	Total P (g kg^{-1})	Alkali-Hydrolysable N (mg kg^{-1})	Olsen-P (mg kg^{-1})	Exchangeable K (mg kg^{-1})	Available Fe (mg kg^{-1})	Available Mn (mg kg^{-1})	Available Cu (mg kg^{-1})	Available Zn (mg kg^{-1})
NF	Control	7.51±0.06	1.49±0.09	0.76±0.02	72.0±4.0	10.4±0.4	177.4±12.6	84.3±6.8	24.7±1.6	6.54±0.39	1.36±0.03
	Duckweed	7.43±0.08	1.67±0.13	0.78±0.01	73.8±6.5	11.5±1.2	211.8±28.5	106.3±12.0	29.9±5.8	6.82±0.37	1.45±0.02
CF	Control	7.44±0.09	1.77±0.04	0.96±0.02	85.1±2.4	29.3±1.1	189.7±13.6	113.7±6.7	24.0±0.7	6.49±0.31	1.38±0.03
	Duckweed	7.10±0.06*	1.80±0.07	0.96±0.04	93.5±1.4*	26.8±1.2	225.1±7.0	110.4±2.9	24.1±0.8	6.61±0.12	1.60±0.15
COF	Control	7.07±0.07	2.19±0.09	1.04±0.04	117.4±5.9	49.1±4.0	223.6±10.7	158.6±10.4	44.6±5.8	7.42±0.36	5.70±0.98
	Duckweed	7.15±0.11	2.11±0.04	1.10±0.01	113.3±6.2	41.6±3.2	234.7±6.0	150.9±7.5	39.7±2.8	7.35±0.23	6.82±0.43
OF	Control	7.01±0.14	2.20±0.05	1.11±0.03	132.0±10.1	57.1±13.2	288.1±18.4	183.1±12.9	51.4±4.4	7.89±0.23	13.48±4.17
	Duckweed	6.86±0.06	2.50±0.03**	1.27±0.03*	162.6±3.7*	66.0±3.0	312.3±3.7	203.4±3.8	64.4±4.8	8.16±0.02	22.69±1.44
ANOVA results											
Fertilizer		**<0.001**	**<0.001**	**<0.001**	**<0.001**	**0.001**	**0.008**	**<0.001**	**0.001**	**0.009**	**<0.001**
Duckweed		0.149	**0.030**	**0.024**	**0.025**	0.992	**0.005**	0.086	0.115	0.286	0.100
Fertilizer×Duckweed		0.328	0.052	0.120	**0.027**	0.255	0.584	0.059	0.052	0.781	0.148

NF, no fertilizer application; CF, chemical fertilizer; COF, two-third of CF and one-third of organic fertilizer; OF, organic fertilizer. Control, no duckweed coverage; Duckweed, duckweed coverage. ** or * indicates significant duckweed effect at $P<0.01$ or $P<0.05$, respectively. ANOVA values in bold indicate significance at $P<0.05$.

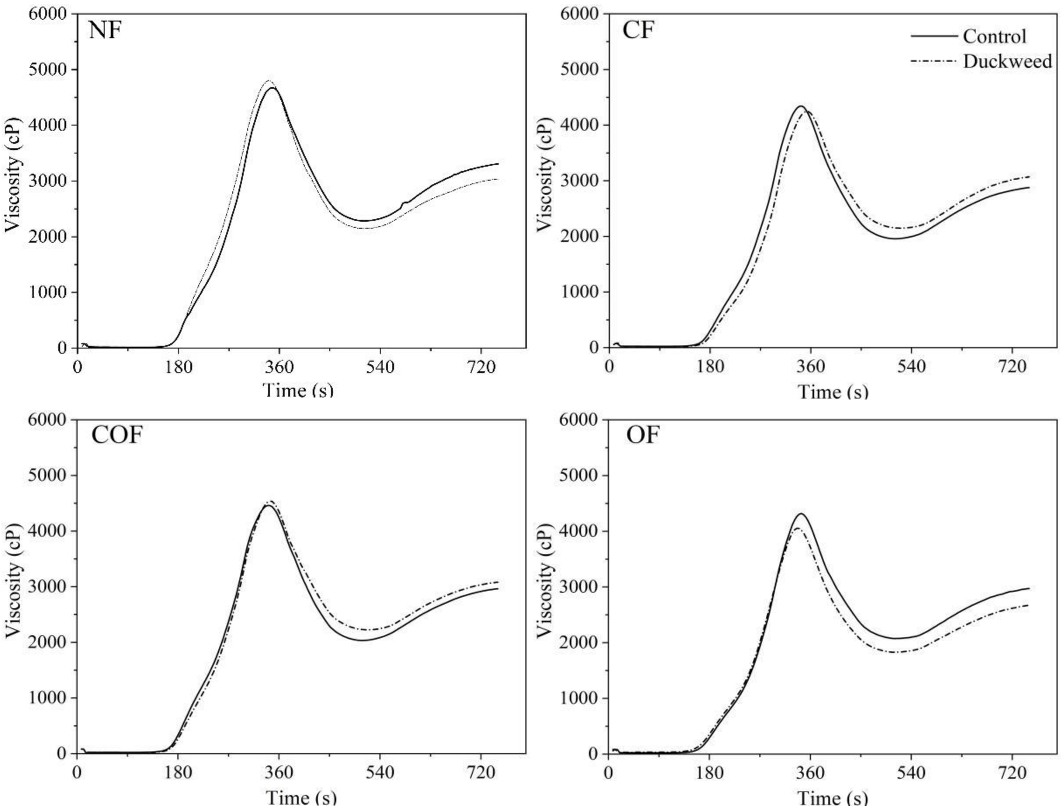

Figure S1. Effects of duckweed coverage on the RVA profiles of milled rice flours under different fertilization conditions. NF, no fertilizer application; CF, chemical fertilizer; COF, two-third of CF and one-third of organic fertilizer; OF, organic fertilizer. Control, no duckweed coverage; Duckweed, duckweed coverage.

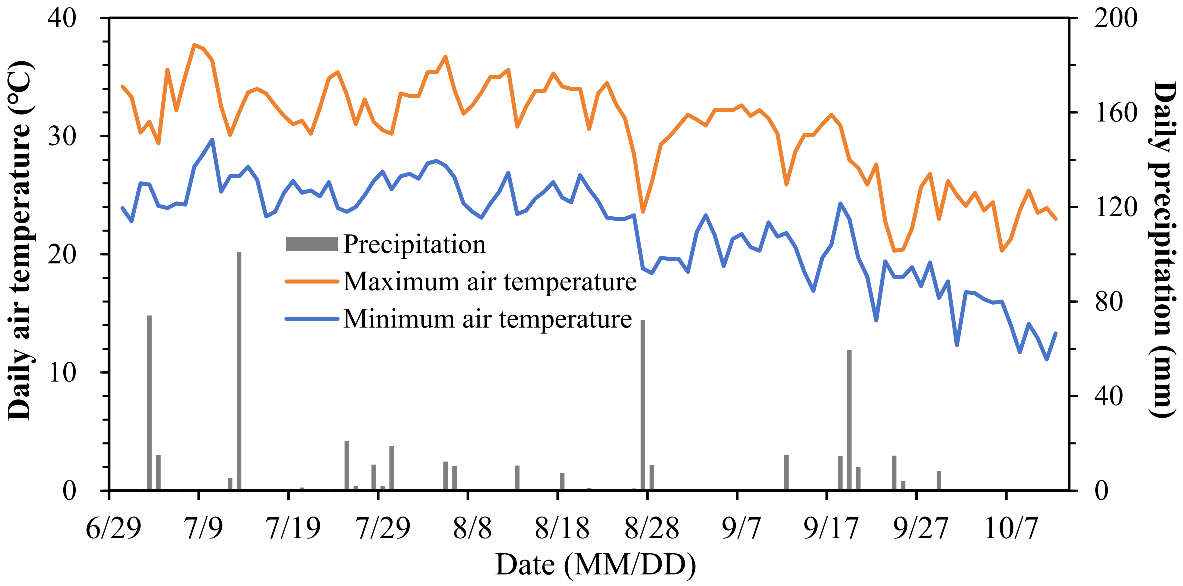

Figure S2. Daily air temperature and precipitation at the experiment site during rice growing season (from transplanting to harvest).
